# Supplementary material for: Investigation of the Potential Effects of Host Genetics and Probiotic Treatment on the Gut Bacterial Community Composition of Aquaculture-raised Pacific Whiteleg Shrimp, Litopenaeus vannamei
Source: Microorganisms. 2019 Jul 26;7(8):217. doi: 10.3390/microorganisms7080217 (PMC6722567; doi:10.3390/microorganisms7080217)
Supplement: Supplementary file 1 [file microorganisms-07-00217-s001.zip › Landsman et al Table 1 Proximate analysis 06272019.docx]

**Table 1.** Proximate analysis for proprietary diet fed to all research tanks.

| **Analyzed Nutrients** | **Units** | **Observed Value** |
| --- | --- | --- |
| Moisture | % | 6.85 |
| Dry Matter | % | 93.15 |
| Protein (crude) | % | 37.50 |
| Fat (crude) | % | 9.54 |
| Fiber (crude) | % | 1.80 |
| Ash | % | 11.40 |
| Digestible Energy | Mcal/lbs | 1.55 |
| Total Digestible Nutrients | % | 77.10 |
| Metabolizable Energy | Mcal/lbs | 1.36 |
| Net Energy (gain) | Mcal/lbs | 0.56 |
| Net Energy (lactation) | Mcal/lbs | 0.81 |
| Net Energy (maint.) | Mcal/lbs | 0.84 |
