# Supplementary material for: Investigation of the Potential Effects of Host Genetics and Probiotic Treatment on the Gut Bacterial Community Composition of Aquaculture-raised Pacific Whiteleg Shrimp, Litopenaeus vannamei
Source: Microorganisms. 2019 Jul 26;7(8):217. doi: 10.3390/microorganisms7080217 (PMC6722567; doi:10.3390/microorganisms7080217)
Supplement: Supplementary file 1 [file microorganisms-07-00217-s001.zip › Landsman et al Table 2 Taxonomy 06182019.docx]

| **Taxonomic group** | **SIS.43** | **SIS.57+** | **SIS.57-** | **SIS.71+** | **SIS.71-** | **OI.43** | **OI.57+** | **OI.57-** | **OI.71+** | **OI.71-** | ***P* values^*^** |
| --- | --- | --- | --- | --- | --- | --- | --- | --- | --- | --- | --- |
| **Proteobacteria** | 77.21 | 80.84 | 43.68 | 87.22 | 64.93 | 62.96 | 79.70 | 47.35 | 73.61 | 56.43 | 0.09470 |
| Rhodobacterales | 47.41^bc^ | 8.79 ^a^ | 11.13 ^a^ | 3.96 ^a^ | 9.63 ^a^ | 12.94 ^a^ | 18.83 ^ac^ | 1.86 ^a^ | 4.43 ^a^ | 6.21 ^a^ | 0.00032 |
| Vibrionales | 27.93 | 71.60 | 31.95 | 82.70 | 54.44 | 47.76 | 59.04 | 45.01 | 63.86 | 48.19 | 0.07790 |
| Other Proteobacteria | 1.86 | 0.46 | 0.60 | 0.57 | 0.86 | 2.26 | 1.83 | 0.48 | 5.32 | 2.03 | ND ^#^ |
| **Bacteroidetes** | 4.80 ^ac^ | 11.99 ^ac^ | 45.98 ^b^ | 8.59 ^ac^ | 30.57 ^bc^ | 4.05 ^a^ | 5.24 ^ac^ | 1.14 ^ac^ | 1.47 ^ac^ | 4.49 ^ac^ | 0.00018 |
| **Verrucomicrobia** | 7.28 ^a^ | 3.66 ^a^ | 8.22 ^ac^ | 2.48 ^a^ | 2.53 ^a^ | 28.80 ^bc^ | 4.87 ^ac^ | 0.51 ^a^ | 1.01 ^a^ | 0.80 ^a^ | 0.00348 |
| **Firmicutes** | 0.47 ^a^ | 0.70 ^a^ | 0.42 ^a^ | 0.83 ^a^ | 0.48 ^a^ | 1.65 ^a^ | 7.56 ^ab^ | 50.13 ^b^ | 22.10 ^ab^ | 37.07 ^ab^ | 0.00159 |
| **Planctomycetes** | 2.97 ^b^ | 0.57 ^ab^ | 0.82 ^ab^ | 0.42 ^a^ | 0.80 ^ab^ | 2.09 ^ab^ | 0.51 ^a^ | 0.34 ^a^ | 0.87 ^ab^ | 0.50 ^a^ | 0.00435 |
| **Saccharibacteria** | 4.87 | 0.41 | 0.13 | 0.07 | 0.22 | 0.06 | 1.02 | 0.02 | 0.25 | 0.09 | 0.13700 |
| **Other Phyla** | 2.17 | 0.42 | 0.54 | 0.28 | 0.39 | 0.18 | 1.02 | 0.49 | 0.56 | 0.39 | ND ^#^ |

**Table 2.** Mean relative abundance (%) of main bacterial taxonomic groups in the intestinal tract of whiteleg shrimp from two genetic lines (SIS or OI), in the presence (+) or absence (-) of probiotic treatment, at three different sampling time points (d43, d57 and d71).

a, b, c. Values statistically different from each other based on Tukey adjustment are distinguished by different superscripts

*determined by ANOVA

#ANOVA was not performed for these groups because they include multiple ranks of the same taxonomic level (i.e. orders or phyla).
