# Supplementary material for: Investigation of the Potential Effects of Host Genetics and Probiotic Treatment on the Gut Bacterial Community Composition of Aquaculture-raised Pacific Whiteleg Shrimp, Litopenaeus vannamei
Source: Microorganisms. 2019 Jul 26;7(8):217. doi: 10.3390/microorganisms7080217 (PMC6722567; doi:10.3390/microorganisms7080217)
Supplement: Supplementary file 1 [file microorganisms-07-00217-s001.zip › Landsman et al Table 3 OTUs 06182019.docx]

| **OTUs** | **SIS.43** | **SIS.57+** | **SIS.57-** | **SIS.71+** | **SIS.71-** | **OI.43** | **OI.57+** | **OI.57-** | **OI.71+** | **OI.71-** | ***P* values^*^** |
| --- | --- | --- | --- | --- | --- | --- | --- | --- | --- | --- | --- |
| **Proteobacteria** |  |  |  |  |  |  |  |  |  |  |  |
| SD_Shr-00002 | 6.89 ^bc^ | 2.24 ^a^ | 3.07 ^ac^ | 0.83 ^a^ | 1.91 ^a^ | 1.73 ^a^ | 2.48 ^a^ | 0.56 ^a^ | 0.59 ^a^ | 0.68 ^a^ | 0.00011 |
| SD_Shr-00004 | 2.38 ^a^ | 7.45 ^ac^ | 7.22 ^ac^ | 24.58 ^bc^ | 33.16 ^b^ | 2.09 ^a^ | 3.88 ^a^ | 3.03 ^a^ | 6.03 ^a^ | 5.63 ^a^ | 6.22E-06 |
| SD_Shr-00006 | 21.91 ^b^ | 2.78 ^a^ | 3.02 ^a^ | 1.00 ^a^ | 0.90 ^a^ | 6.82 ^a^ | 4.54 ^a^ | 0.39 ^a^ | 1.08 ^a^ | 2.92 ^a^ | 0.000824 |
| SD_Shr-00010 | 22.02 | 58.69 | 21.32 | 50.01 | 14.78 | 40.06 | 49.16 | 38.13 | 48.11 | 34.66 | 0.114 |
| **Firmicutes** |  |  |  |  |  |  |  |  |  |  |  |
| SD_Shr-00003 | 0.39 ^a^ | 0.52 ^a^ | 0.33 ^a^ | 0.64 ^a^ | 0.38 ^a^ | 1.56 ^a^ | 2.81 ^a^ | 49.00 ^b^ | 21.44 ^ab^ | 36.14 ^ab^ | 0.00161 |
| **Bacteroidetes** |  |  |  |  |  |  |  |  |  |  |  |
| SD_Shr-00097 | 1.95 ^a^ | 8.13 ^a^ | 40.11 ^bc^ | 2.11 ^a^ | 25.68 ^ac^ | 3.13 ^a^ | 2.46 ^a^ | 0.68 ^a^ | 0.60 ^a^ | 2.30 ^a^ | 0.000345 |
| **Verrucomicrobia** |  |  |  |  |  |  |  |  |  |  |  |
| SD_Shr-00098 | 2.50 ^a^ | 1.26 ^a^ | 6.43 ^ab^ | 0.42 ^a^ | 0.83 ^a^ | 25.27 ^b^ | 4.18 ^ab^ | 0.41 ^a^ | 0.72 ^a^ | 0.51 ^a^ | 0.00626 |

**Table 3.** Mean relative abundance (%) of main bacterial Operational Taxonomic Units in the intestinal tract of whiteleg shrimp from two genetic lines (SIS or OI), in the presence (+) or absence (-) of probiotic treatment, at three different sampling time points (d43, d57 and d71).

a, b, c. Values statistically different from each other based on Tukey adjustment are distinguished by different superscripts

*determined by ANOVA

#ANOVA was not performed for these groups because they include multiple ranks of the same taxonomic level (i.e. orders or phyla).
