# Supplementary material for: Investigation of the Potential Effects of Host Genetics and Probiotic Treatment on the Gut Bacterial Community Composition of Aquaculture-raised Pacific Whiteleg Shrimp, Litopenaeus vannamei
Source: Microorganisms. 2019 Jul 26;7(8):217. doi: 10.3390/microorganisms7080217 (PMC6722567; doi:10.3390/microorganisms7080217)
Supplement: Supplementary file 1 [file microorganisms-07-00217-s001.zip › Landsman et al Supplementary data file 06272019.docx]

**Table S1.** Daily total ammonia nitrogen results from Hach spectrophotometer analysis.

| **Shrimp Age** | **SIS.ST1** | **SIS.ST2** | **SIS.ST3** | **SIS.STA** | **SIS.STB** | **SIS.STC** | **OI.ST1** | **OI.ST2** | **OI.ST3** | **OI.STA** | **OI.STB** | **OI.STC** |
| --- | --- | --- | --- | --- | --- | --- | --- | --- | --- | --- | --- | --- |
| 42 | 0.21 | 0.24 | 0.17 | 0.22 | 0.18 | 0.20 | 0.33 | 0.63 | 0.74 | 0.31 | 2.40 | 2.84 |
| 43 | 0.20 | 0.26 | 0.16 | 0.24 | 0.27 | 0.25 | 1.80 | 1.38 | 1.60 | 0.12 | 1.00 | 0.84 |
| 44 | 0.22 | 0.22 | 0.24 | 0.29 | 0.17 | 0.35 | 0.38 | 0.88 | 0.72 | 0.35 | 1.36 | 1.45 |
| 45 | 0.22 | 0.23 | 0.23 | 0.19 | 0.29 | 0.20 | 0.25 | 0.92 | 0.62 | 0.26 | 0.72 | 0.98 |
| 46 | 0.28 | 0.32 | 0.30 | 0.24 | 0.30 | 0.38 | 0.52 | 0.78 | 0.84 | 0.18 | 0.66 | 0.78 |
| 47 | 0.26 | 0.25 | 0.22 | 0.25 | 0.29 | 0.33 | 0.26 | 0.61 | 0.80 | 0.09 | 0.50 | 0.56 |
| 48 | 0.22 | 0.23 | 0.22 | 0.21 | 0.23 | 0.24 | 0.18 | 0.62 | 0.80 | 0.05 | 0.24 | 0.42 |
| 49 | 0.21 | 0.20 | 0.25 | 0.19 | 0.24 | 0.32 | 0.12 | 0.65 | 0.30 | 0.13 | 0.59 | 0.16 |
| 50 | 0.28 | 0.15 | 0.24 | 0.27 | 0.25 | 0.31 | 0.11 | 0.22 | 0.37 | 0.10 | 0.45 | 0.38 |
| 51 | 0.24 | 0.24 | 0.22 | 0.24 | 0.25 | 0.31 | 0.10 | 0.12 | 0.07 | 0.03 | 0.09 | 0.08 |
| 52 | 0.15 | 0.29 | 0.33 | 0.21 | 0.37 | 0.33 | 0.22 | 0.25 | 0.18 | 0.15 | 0.47 | 0.52 |
| 53 | 0.17 | 0.29 | 0.32 | 0.22 | 0.41 | 0.38 | 0.21 | 0.10 | 0.13 | 0.23 | 0.19 | 0.10 |
| 54 | 0.20 | 0.29 | 0.31 | 0.24 | 0.46 | 0.42 | 0.26 | 0.22 | 0.31 | 0.24 | 0.30 | 0.38 |
| 55 | 0.32 | 0.23 | 0.28 | 0.15 | 0.40 | 0.42 | 0.37 | 0.37 | 0.35 | 0.32 | 0.25 | 0.26 |
| 56 | 0.19 | 0.28 | 0.24 | 0.14 | 0.24 | 0.29 | 0.36 | 0.31 | 0.28 | 0.27 | 0.16 | 0.43 |
| 57 | 0.21 | 0.15 | 0.25 | 0.24 | 0.27 | 0.17 | 0.41 | 0.32 | 0.32 | 0.32 | 0.44 | 0.36 |
| 58 | 0.28 | 0.29 | 0.26 | 0.22 | 0.23 | 0.33 | 0.33 | 0.33 | 0.34 | 0.31 | 0.21 | 0.17 |
| 59 | 0.26 | 0.31 | 0.26 | 0.29 | 0.30 | 0.24 | 0.37 | 0.34 | 0.32 | 0.35 | 0.47 | 0.36 |
| 60 | 0.27 | 0.31 | 0.33 | 0.31 | 0.31 | 0.33 | 0.27 | 0.31 | 0.35 | 0.40 | 0.46 | 0.40 |
| 61 | 0.25 | 0.29 | 0.30 | 0.24 | 0.30 | 0.29 | 0.34 | 0.27 | 0.27 | 0.37 | 0.39 | 0.31 |
| 62 | 0.23 | 0.27 | 0.26 | 0.18 | 0.28 | 0.25 | 0.21 | 0.30 | 0.19 | 0.30 | 0.31 | 0.21 |
| 63 | 0.11 | 0.12 | 0.18 | 0.10 | 0.18 | 0.08 | 0.09 | 0.28 | 0.33 | 0.18 | 0.31 | 0.29 |
| 64 | 0.16 | 0.17 | 0.24 | 0.21 | 0.19 | 0.18 | 0.35 | 0.29 | 0.37 | 0.34 | 0.43 | 0.58 |
| 65 | 0.26 | 0.23 | 0.28 | 0.27 | 0.35 | 0.34 | 0.46 | 0.28 | 0.35 | 0.43 | 0.43 | 0.62 |
| 66 | 0.16 | 0.15 | 0.19 | 0.15 | 0.22 | 0.14 | 0.50 | 0.36 | 0.38 | 0.48 | 0.58 | 0.52 |
| 67 | 0.28 | 0.32 | 0.32 | 0.27 | 0.52 | 0.31 | 0.29 | 0.22 | 0.32 | 0.28 | 0.34 | 0.31 |
| 68 | 0.34 | 0.36 | 0.40 | 0.42 | 0.39 | 0.32 | 0.42 | 0.30 | 0.26 | 0.46 | 0.46 | 0.50 |
| 69 | 0.32 | 0.26 | 0.31 | 0.32 | 0.42 | 0.37 | 0.36 | 0.38 | 0.38 | 0.48 | 0.70 | 0.52 |
| 70 | 0.22 | 0.33 | 0.38 | 0.25 | 0.36 | 0.29 | 0.30 | 0.20 | 0.25 | 0.30 | 0.40 | 0.35 |
| 71 | 0.42 | 0.22 | 0.52 | 0.24 | 0.29 | 0.26 | 0.36 | 0.28 | 0.28 | 0.41 | 0.36 | 0.24 |
| 72 | 0.36 | 0.43 | 0.66 | 0.35 | 0.39 | 0.21 | 0.08 | 0.08 | 0.08 | 0.11 | 0.08 | 0.13 |

**Table S2.** Monday, Wednesday, Friday nitrite nitrogen results from Hach spectrophotometer analysis.

| **Shrimp Age** | **SIS.ST1** | **SIS.ST2** | **SIS.ST3** | **SIS.STA** | **SIS.STB** | **SIS.STC** | **OI.ST1** | **OI.ST2** | **OI.ST3** | **OI.STA** | **OI.STB** | **OI.STC** |
| --- | --- | --- | --- | --- | --- | --- | --- | --- | --- | --- | --- | --- |
| 42 | 0.14 | 0.20 | 0.16 | 0.11 | 0.19 | 0.31 | 3.31 | 3.86 | 2.73 | 0.25 | 3.92 | 3.01 |
| 45 | 0.15 | 0.15 | 0.15 | 0.16 | 0.25 | 0.25 | 2.60 | 4.11 | 2.00 | 0.25 | 4.96 | 3.96 |
| 48 | 0.13 | 0.14 | 0.18 | 0.14 | 0.21 | 0.24 | 0.88 | 1.45 | 2.01 | 0.22 | 3.95 | 4.60 |
| 51 | 0.20 | 0.16 | 0.27 | 0.18 | 0.23 | 0.32 | 0.58 | 0.64 | 0.42 | 0.18 | 3.96 | 3.89 |
| 54 | 0.21 | 0.18 | 0.33 | 0.18 | 0.28 | 0.33 | 0.63 | 0.51 | 0.51 | 0.23 | 4.05 | 4.65 |
| 57 | 0.19 | 0.22 | 0.30 | 0.21 | 0.42 | 0.44 | 0.75 | 0.41 | 0.47 | 0.28 | 0.90 | 2.01 |
| 60 | 0.22 | 0.29 | 0.30 | 0.25 | 0.41 | 0.49 | 0.62 | 0.43 | 0.36 | 0.28 | 1.08 | 1.63 |
| 63 | 0.22 | 0.35 | 0.40 | 0.19 | 0.55 | 0.66 | 0.93 | 0.19 | 0.20 | 0.14 | 0.68 | 0.68 |
| 66 | 0.37 | 0.47 | 0.45 | 0.26 | 0.52 | 0.60 | 0.80 | 0.47 | 0.40 | 0.36 | 1.96 | 1.95 |
| 69 | 0.38 | 0.46 | 0.51 | 0.35 | 0.60 | 0.71 | 0.71 | 0.55 | 0.36 | 0.32 | 3.84 | 1.60 |
| 72 | 0.40 | 0.59 | 0.51 | 0.30 | 0.61 | 0.56 | 0.77 | 0.65 | 0.44 | 0.48 | 2.71 | 0.99 |

**Table S3.** Monday, Wednesday, Friday nitrate nitrogen results from Hach spectrophotometer analysis.

| **Shrimp Age** | **SIS.ST1** | **SIS.ST2** | **SIS.ST3** | **SIS.STA** | **SIS.STB** | **SIS.STC** | **OI.ST1** | **OI.ST2** | **OI.ST3** | **OI.STA** | **OI.STB** | **OI.STC** |
| --- | --- | --- | --- | --- | --- | --- | --- | --- | --- | --- | --- | --- |
| 42 | 6.55 | 6.20 | 6.37 | 8.58 | 6.90 | 6.02 | 6.28 | 5.14 | 5.58 | 6.55 | 7.79 | 4.49 |
| 45 | 8.38 | 8.14 | 8.14 | 4.96 | 10.03 | 7.91 | 6.81 | 6.28 | 6.99 | 6.55 | 6.23 | 4.38 |
| 48 | 5.08 | 7.67 | 7.25 | 10.74 | 11.44 | 12.98 | 7.08 | 7.67 | 4.34 | 5.84 | 5.80 | 6.85 |
| 51 | 13.21 | 7.79 | 13.68 | 12.74 | 11.13 | 10.50 | 9.44 | 6.72 | 7.67 | 7.61 | 6.38 | 7.08 |
| 54 | 13.80 | 11.80 | 15.81 | 14.86 | 14.98 | 15.10 | 15.57 | 11.56 | 12.74 | 10.15 | 16.87 | 15.57 |
| 57 | 8.14 | 7.32 | 12.74 | 11.75 | 14.57 | 11.44 | 7.52 | 6.73 | 10.85 | 7.61 | 14.47 | 6.90 |
| 60 | 8.05 | 10.85 | 7.87 | 13.21 | 15.45 | 13.96 | 9.11 | 15.59 | 11.05 | 11.93 | 4.60 | 3.84 |
| 63 | 12.98 | 9.37 | 12.98 | 11.57 | 16.28 | 12.50 | 10.38 | 8.22 | 16.63 | 11.92 | 12.03 | 10.97 |
| 66 | 7.34 | 8.05 | 12.15 | 15.92 | 20.29 | 16.39 | 14.16 | 12.63 | 19.34 | 26.06 | 13.69 | 17.22 |
| 69 | 15.92 | 16.99 | 22.53 | 19.58 | 20.76 | 23.23 | 16.16 | 20.40 | 22.76 | 20.17 | 22.41 | 13.09 |
| 72 | 19.35 | 10.86 | 14.61 | 17.23 | 24.90 | 17.23 | 9.71 | 11.43 | 14.29 | 16.41 | 13.47 | 12.74 |

**Table S4.** Daily alkalinity results from Hach spectrophotometer analysis.

| **Shrimp Age** | **SIS.ST1** | **SIS.ST2** | **SIS.ST3** | **SIS.STA** | **SIS.STB** | **SIS.STC** | **OI.ST1** | **OI.ST2** | **OI.ST3** | **OI.STA** | **OI.STB** | **OI.STC** |
| --- | --- | --- | --- | --- | --- | --- | --- | --- | --- | --- | --- | --- |
| 42 | 200 | 197 | 200 | 209 | 191 | 198 | 205 | 213 | 195 | 177 | 234 | 224 |
| 43 | 189 | 204 | 182 | 199 | 193 | 186 | 158 | 220 | 211 | 174 | 188 | 182 |
| 44 | 197 | 206 | 188 | 200 | 190 | 194 | 213 | 202 | 200 | 172 | 216 | 224 |
| 45 | 190 | 189 | 191 | 197 | 195 | 198 | 200 | 194 | 195 | 183 | 228 | 207 |
| 46 | 197 | 205 | 188 | 203 | 203 | 192 | 188 | 196 | 197 | 182 | 207 | 209 |
| 47 | 193 | 192 | 195 | 191 | 194 | 194 | 170 | 160 | 160 | 160 | 170 | 160 |
| 48 | 195 | 198 | 188 | 196 | 188 | 196 | 207 | 203 | 195 | 188 | 210 | 214 |
| 49 | 204 | 202 | 194 | 189 | 194 | 200 | 294 | 227 | 233 | 196 | 205 | 202 |
| 50 | 190 | 203 | 198 | 186 | 210 | 181 | 200 | 204 | 184 | 192 | 183 | 195 |
| 51 | 186 | 176 | 201 | 174 | 207 | 175 | 219 | 210 | 206 | 186 | 187 | 198 |
| 52 | 185 | 194 | 180 | 181 | 205 | 184 | 197 | 207 | 207 | 200 | 179 | 180 |
| 53 | 180 | 200 | 200 | 200 | 200 | 200 | 179 | 204 | 192 | 202 | 180 | 191 |
| 54 | 190 | 199 | 184 | 182 | 216 | 196 | 172 | 186 | 177 | 204 | 165 | 177 |
| 55 | 199 | 201 | 182 | 190 | 219 | 195 | 194 | 174 | 183 | 214 | 177 | 186 |
| 56 | 194 | 215 | 190 | 193 | 233 | 203 | 188 | 198 | 199 | 225 | 173 | 195 |
| 57 | 201 | 201 | 190 | 193 | 218 | 205 | 177 | 198 | 203 | 209 | 174 | 199 |
| 58 | 191 | 208 | 169 | 188 | 237 | 204 | 169 | 179 | 182 | 204 | 160 | 156 |
| 59 | 190 | 199 | 183 | 188 | 240 | 199 | 168 | 174 | 189 | 210 | 175 | 171 |
| 60 | 184 | 207 | 168 | 197 | 251 | 194 | 183 | 186 | 175 | 212 | 177 | 181 |
| 61 | 180 | 200 | 160 | 200 | 200 | 200 | 169 | 179 | 168 | 211 | 172 | 179 |
| 62 | 167 | 179 | 181 | 178 | 236 | 195 | 170 | 177 | 185 | 206 | 172 | 188 |
| 63 | 196 | 190 | 185 | 189 | 251 | 219 | 187 | 195 | 210 | 228 | 188 | 208 |
| 64 | 182 | 168 | 173 | 177 | 256 | 203 | 182 | 192 | 195 | 218 | 179 | 213 |
| 65 | 179 | 179 | 187 | 181 | 232 | 201 | 177 | 183 | 195 | 222 | 176 | 191 |
| 66 | 179 | 182 | 173 | 178 | 243 | 197 | 184 | 178 | 187 | 209 | 180 | 208 |
| 67 | 181 | 168 | 185 | 179 | 256 | 212 | 173 | 187 | 186 | 199 | 172 | 193 |
| 68 | 185 | 200 | 201 | 195 | 266 | 220 | 179 | 180 | 179 | 195 | 195 | 201 |
| 69 | 182 | 189 | 189 | 185 | 258 | 216 | 169 | 179 | 202 | 200 | 186 | 197 |
| 70 | 188 | 187 | 207 | 198 | 284 | 212 | 177 | 173 | 185 | 184 | 159 | 176 |
| 71 | 165 | 169 | 196 | 200 | 271 | 225 | 183 | 182 | 190 | 193 | 190 | 211 |
| 72 | 189 | 177 | 188 | 201 | 275 | 225 | 189 | 215 | 205 | 208 | 193 | 208 |

**Table S5.** Daily pH results from hand-held YSI meter.

| **Shrimp Age** | **SIS.ST1** | **SIS.ST2** | **SIS.ST3** | **SIS.STA** | **SIS.STB** | **SIS.STC** | **OI.ST1** | **OI.ST2** | **OI.ST3** | **OI.STA** | **OI.STB** | **OI.STC** |
| --- | --- | --- | --- | --- | --- | --- | --- | --- | --- | --- | --- | --- |
| 42 | 7.56 | 7.56 | 7.62 | 7.67 | 7.57 | 7.59 | 7.93 | 7.74 | 7.77 | 7.65 | 7.90 | 7.70 |
| 43 | 7.46 | 7.41 | 7.60 | 7.58 | 7.45 | 7.54 | 7.81 | 7.76 | 7.76 | 7.76 | 7.89 | 7.89 |
| 44 | 7.50 | 7.54 | 7.69 | 7.66 | 7.47 | 7.59 | 7.86 | 7.79 | 7.81 | 7.65 | 7.86 | 7.77 |
| 45 | 7.35 | 7.38 | 7.49 | 7.46 | 7.32 | 7.53 | 7.79 | 7.67 | 7.71 | 7.61 | 7.81 | 7.72 |
| 46 | 7.45 | 7.42 | 7.50 | 7.48 | 7.35 | 7.45 | 7.80 | 7.67 | 7.73 | 7.57 | 7.81 | 7.69 |
| 47 | 7.44 | 7.41 | 7.46 | 7.41 | 7.42 | 7.43 | 7.23 | 7.24 | 7.31 | 7.18 | 7.35 | 7.32 |
| 48 | 7.75 | 7.62 | 7.64 | 7.62 | 7.54 | 7.55 | 7.77 | 7.67 | 7.74 | 7.61 | 7.77 | 7.64 |
| 49 | 7.33 | 7.36 | 7.40 | 7.44 | 7.38 | 7.40 | 7.58 | 7.65 | 7.73 | 7.59 | 7.72 | 7.70 |
| 50 | 7.59 | 7.50 | 7.52 | 7.52 | 7.30 | 7.45 | 7.64 | 7.57 | 7.63 | 7.51 | 7.61 | 7.58 |
| 51 | 7.49 | 7.52 | 7.55 | 7.54 | 7.51 | 7.51 | 7.51 | 7.52 | 7.59 | 7.27 | 7.59 | 7.50 |
| 52 | 7.44 | 7.36 | 7.37 | 7.33 | 7.25 | 7.37 | 7.54 | 7.50 | 7.52 | 7.30 | 7.55 | 7.42 |
| 53 | 7.25 | 7.26 | 7.36 | 7.40 | 7.28 | 7.33 | 7.55 | 7.62 | 7.61 | 7.36 | 7.49 | 7.40 |
| 54 | 7.37 | 7.42 | 7.47 | 7.46 | 7.31 | 7.31 | 7.37 | 7.37 | 7.35 | 7.29 | 7.42 | 7.35 |
| 55 | 7.41 | 7.35 | 7.45 | 7.36 | 7.22 | 7.33 | 7.43 | 7.39 | 7.29 | 7.30 | 7.40 | 7.25 |
| 56 | 7.34 | 7.39 | 7.43 | 7.39 | 7.32 | 7.38 | 7.42 | 7.44 | 7.29 | 7.21 | 7.46 | 7.38 |
| 57 | 7.55 | 7.53 | 7.45 | 7.40 | 7.36 | 7.47 | 7.50 | 7.59 | 7.59 | 7.40 | 7.52 | 7.42 |
| 58 | 7.50 | 7.48 | 7.44 | 7.38 | 7.33 | 7.40 | 7.46 | 7.39 | 7.49 | 7.24 | 7.78 | 7.78 |
| 59 | 7.43 | 7.45 | 7.46 | 7.40 | 7.32 | 7.39 | 7.38 | 7.35 | 7.40 | 7.30 | 7.31 | 7.31 |
| 60 | 7.40 | 7.41 | 7.40 | 7.35 | 7.33 | 7.36 | 7.40 | 7.34 | 7.24 | 7.25 | 7.34 | 7.28 |
| 61 | 7.34 | 7.41 | 7.36 | 7.39 | 7.35 | 7.34 | 7.44 | 7.42 | 7.32 | 7.31 | 7.40 | 7.37 |
| 62 | 7.38 | 7.44 | 7.48 | 7.46 | 7.41 | 7.44 | 7.52 | 7.51 | 7.45 | 7.40 | 7.47 | 7.40 |
| 63 | 7.30 | 7.37 | 7.40 | 7.41 | 7.34 | 7.37 | 7.46 | 7.38 | 7.40 | 7.36 | 7.39 | 7.34 |
| 64 | 7.54 | 7.51 | 7.50 | 7.35 | 7.35 | 7.41 | 7.25 | 7.30 | 7.20 | 7.29 | 7.37 | 7.27 |
| 65 | 7.48 | 7.43 | 7.40 | 7.48 | 7.27 | 7.34 | 7.34 | 7.38 | 7.36 | 7.29 | 7.35 | 7.33 |
| 66 | 7.33 | 7.40 | 7.37 | 7.34 | 7.38 | 7.34 | 7.48 | 7.50 | 7.53 | 7.44 | 7.54 | 7.43 |
| 67 | 7.39 | 7.35 | 7.37 | 7.34 | 7.24 | 7.29 | 7.37 | 7.38 | 7.37 | 7.31 | 7.34 | 7.28 |
| 68 | 7.34 | 7.35 | 7.36 | 7.32 | 7.24 | 7.34 | 7.46 | 7.47 | 7.38 | 7.34 | 7.42 | 7.29 |
| 69 | 7.33 | 7.32 | 7.34 | 7.30 | 7.21 | 7.31 | 7.41 | 7.35 | 7.41 | 7.33 | 7.42 | 7.21 |
| 70 | 7.43 | 7.45 | 7.44 | 7.41 | 7.41 | 7.39 | 7.41 | 7.38 | 7.39 | 7.28 | 7.32 | 7.24 |
| 71 | 7.33 | 7.39 | 7.35 | 7.31 | 7.34 | 7.35 | 7.30 | 7.14 | 7.24 | 7.18 | 7.38 | 7.50 |
| 72 | 7.27 | 7.29 | 7.34 | 7.35 | 7.40 | 7.30 | 7.34 | 7.42 | 7.21 | 7.31 | 7.39 | 7.29 |

**Table S6.** Weekly inert vibrio colony forming unit plate results from independent lab RTI.

| **Shrimp Age** | **SIS.ST1** | **SIS.ST2** | **SIS.ST3** | **SIS.STA** | **SIS.STB** | **SIS.STC** | **OI.ST1** | **OI.ST2** | **OI.ST3** | **OI.STA** | **OI.STB** | **OI.STC** |
| --- | --- | --- | --- | --- | --- | --- | --- | --- | --- | --- | --- | --- |
| 39 | 1300 | 590 | 370 | 1200 | 380 | 220 | 8300 | 3800 | 920 | 3200 | 2200 | 1100 |
| 46 | 2800 | 1400 | 2300 | 3000 | 2600 | 2800 | 5460 | 4740 | 4020 | 2400 | 4860 | 11400 |
| 53 | 8000 | 1500 | 1600 | 7100 | 2300 | 26000 | 8800 | 2300 | 4100 | 7000 | 3600 | 3900 |
| 60 | 7100 | 2100 | 9200 | 6100 | 2800 | 2100 | 2300 | 800 | 2400 | 2600 | 15000 | 3800 |
| 67 | 11000 | 7000 | 11000 | 8100 | 6100 | 7500 | 1800 | 4600 | 7200 | 2600 | 15000 | 3400 |

**Table S7.** Weekly total heterotrophic colony forming unit plate results from independent lab RTI.

| **Shrimp Age** | **SIS.ST1** | **SIS.ST2** | **SIS.ST3** | **SIS.STA** | **SIS.STB** | **SIS.STC** | **OI.ST1** | **OI.ST2** | **OI.ST3** | **OI.STA** | **OI.STB** | **OI.STC** |
| --- | --- | --- | --- | --- | --- | --- | --- | --- | --- | --- | --- | --- |
| 39 | 410000 | 300000 | 110000 | 170000 | 180000 | 140000 | 200000 | 69000 | 190000 | 200000 | 470000 | 1000000 |
| 46 | 290000 | 270000 | 280000 | 190000 | 160000 | 350000 | 159000 | 117000 | 107000 | 20000 | 310000 | 370000 |
| 53 | 120000 | 140000 | 610000 | 250000 | 390000 | 660000 | 160000 | 50000 | 160000 | 30000 | 290000 | 130000 |
| 60 | 44000 | 490000 | 69000 | 61000 | 75000 | 27000 | 46000 | 40000 | 65000 | 72000 | 16000 | 130000 |
| 67 | 140000 | 230000 | 140000 | 630000 | 290000 | 270000 | 48000 | 63000 | 51000 | 58000 | 190000 | 92000 |

**Table S8.** Weekly Pediococcus colony forming unit plate results from independent lab RTI.

| **Shrimp Age** | **SIS.ST1** | **SIS.ST2** | **SIS.ST3** | **SIS.STA** | **SIS.STB** | **SIS.STC** | **OI.ST1** | **OI.ST2** | **OI.ST3** | **OI.STA** | **OI.STB** | **OI.STC** |
| --- | --- | --- | --- | --- | --- | --- | --- | --- | --- | --- | --- | --- |
| 46 | 100 | 100 | 0 | 0 | 100 | 0 | 0 | 100 | 100 | 0 | 600 | 400 |
| 53 | 300 | 0 | 0 | 0 | 4800 | 0 | 3000 | 800 | 0 | 1200 | 0 | 500 |
| 60 | 0 | 100 | 0 | 0 | 0 | 0 | 400 | 200 | 5000 | 0 | 0 | 200 |
| 67 | 0 | 5600 | 0 | 0 | 0 | 0 | 300 | 0 | 0 | 0 | 0 | 0 |

**Table S9.** Weekly animal health exam results and calculations.

| **SIS.ST1** | **D43** | **D50** | **D57** | **D64** | **D71** |
| --- | --- | --- | --- | --- | --- |
| Weight (g) | 1.972 | 3.614 | 4.654 | 8.570 | 10.618 |
| Length (cm) | 5.560 | 6.620 | 7.520 | 9.200 | 9.240 |
| Hepatopancreas weight (g) | 0.107 | 0.222 | 0.248 | 0.273 | 0.477 |
| Antenna Length (cm) | 6.710 | 4.270 | 4.550 | 4.440 | 3.590 |
| MCF | 1.147 | 1.246 | 1.094 | 1.101 | 1.346 |
| HSI | 0.054 | 0.061 | 0.053 | 0.032 | 0.045 |
| Shrimp-Antenna Length Ratio | 1.207 | 0.645 | 0.605 | 0.483 | 0.389 |
|  |  |  |  |  |  |
| **SIS.ST2** | **D43** | **D50** | **D57** | **D64** | **D71** |
| Weight (g) | 3.324 | 3.430 | 6.922 | 8.870 | 10.682 |
| Length (cm) | 6.800 | 6.400 | 8.500 | 9.300 | 9.840 |
| Hepatopancreas weight (g) | 0.165 | 0.204 | 0.399 | 0.439 | 0.487 |
| Antenna Length (cm) | 7.830 | 4.180 | 6.270 | 4.770 | 5.610 |
| MCF | 1.057 | 1.308 | 1.127 | 1.103 | 1.121 |
| HSI | 0.050 | 0.059 | 0.058 | 0.049 | 0.046 |
| Shrimp-Antenna Length Ratio | 1.151 | 0.653 | 0.738 | 0.513 | 0.570 |
|  |  |  |  |  |  |
| **SIS.ST3** | **D43** | **D50** | **D57** | **D64** | **D71** |
| Weight (g) | 2.846 | 3.886 | 5.512 | 6.854 | 11.490 |
| Length (cm) | 6.080 | 6.900 | 7.860 | 8.720 | 9.760 |
| Hepatopancreas weight (g) | 0.166 | 0.202 | 0.285 | 0.362 | 0.607 |
| Antenna Length (cm) | 7.440 | 7.070 | 5.940 | 5.240 | 6.400 |
| MCF | 1.266 | 1.183 | 1.135 | 1.034 | 1.236 |
| HSI | 0.058 | 0.052 | 0.052 | 0.053 | 0.053 |
| Shrimp-Antenna Length Ratio | 1.224 | 1.025 | 0.756 | 0.601 | 0.656 |

| **SIS.STA** | **D43** | **D50** | **D57** | **D64** | **D71** |
| --- | --- | --- | --- | --- | --- |
| Weight (g) | 3.168 | 4.210 | 6.962 | 8.406 | 11.358 |
| Length (cm) | 6.520 | 6.900 | 8.040 | 9.380 | 10.200 |
| Hepatopancreas weight (g) | 0.161 | 0.268 | 0.370 | 0.422 | 0.500 |
| Antenna Length (cm) | 8.820 | 6.530 | 5.020 | 4.620 | 5.440 |
| MCF | 1.143 | 1.282 | 1.340 | 1.019 | 1.070 |
| HSI | 0.051 | 0.064 | 0.053 | 0.050 | 0.044 |
| Shrimp-Antenna Length Ratio | 1.353 | 0.946 | 0.624 | 0.493 | 0.533 |
|  |  |  |  |  |  |
| **SIS.STB** | **D43** | **D50** | **D57** | **D64** | **D71** |
| Weight (g) | 4.138 | 5.008 | 6.860 | 10.174 | 11.862 |
| Length (cm) | 7.320 | 7.260 | 7.800 | 9.880 | 10.180 |
| Hepatopancreas weight (g) | 0.188 | 0.277 | 0.316 | 0.474 | 0.540 |
| Antenna Length (cm) | 8.950 | 6.590 | 7.310 | 5.020 | 6.050 |
| MCF | 1.055 | 1.309 | 1.446 | 1.055 | 1.124 |
| HSI | 0.045 | 0.055 | 0.046 | 0.047 | 0.046 |
| Shrimp-Antenna Length Ratio | 1.223 | 0.908 | 0.937 | 0.508 | 0.594 |
|  |  |  |  |  |  |
| **SIS.STC** | **D43** | **D50** | **D57** | **D64** | **D71** |
| Weight (g) | 2.866 | 3.798 | 6.438 | 7.310 | 10.500 |
| Length (cm) | 6.400 | 6.580 | 7.780 | 8.880 | 9.740 |
| Hepatopancreas weight (g) | 0.095 | 0.244 | 0.323 | 0.353 | 0.547 |
| Antenna Length (cm) | 6.680 | 7.350 | 4.810 | 4.860 | 6.240 |
| MCF | 1.093 | 1.333 | 1.367 | 1.044 | 1.136 |
| HSI | 0.033 | 0.064 | 0.050 | 0.048 | 0.052 |
| Shrimp-Antenna Length Ratio | 1.044 | 1.117 | 0.618 | 0.547 | 0.641 |

| **OI.ST1** | **D43** | **D50** | **D57** | **D64** | **D71** |
| --- | --- | --- | --- | --- | --- |
| Weight (g) | 2.326 | 4.744 | 5.386 | 6.672 | 10.668 |
| Length (cm) | 5.740 | 7.580 | 7.580 | 8.480 | 9.960 |
| Hepatopancreas weight (g) | 0.155 | 0.300 | 0.315 | 0.374 | 0.568 |
| Antenna Length (cm) | 6.830 | 6.760 | 6.040 | 3.540 | 4.740 |
| MCF | 1.230 | 1.089 | 1.237 | 1.094 | 1.080 |
| HSI | 0.067 | 0.063 | 0.059 | 0.056 | 0.053 |
| Shrimp-Antenna Length Ratio | 1.190 | 0.892 | 0.797 | 0.417 | 0.476 |
|  |  |  |  |  |  |
| **OI.ST2** | **D43** | **D50** | **D57** | **D64** | **D71** |
| Weight (g) | 2.212 | 3.160 | 5.092 | 6.726 | 10.142 |
| Length (cm) | 5.920 | 6.560 | 7.380 | 8.580 | 9.680 |
| Hepatopancreas weight (g) | 0.132 | 0.192 | 0.311 | 0.392 | 0.592 |
| Antenna Length (cm) | 5.270 | 4.920 | 4.390 | 5.530 | 3.370 |
| MCF | 1.066 | 1.119 | 1.267 | 1.065 | 1.118 |
| HSI | 0.060 | 0.061 | 0.061 | 0.058 | 0.058 |
| Shrimp-Antenna Length Ratio | 0.890 | 0.750 | 0.595 | 0.645 | 0.348 |
|  |  |  |  |  |  |
| **OI.ST3** | **D43** | **D50** | **D57** | **D64** | **D71** |
| Weight (g) | 2.584 | 3.842 | 6.636 | 9.884 | 10.324 |
| Length (cm) | 6.120 | 6.780 | 8.400 | 9.800 | 9.560 |
| Hepatopancreas weight (g) | 0.154 | 0.225 | 0.421 | 0.525 | 0.507 |
| Antenna Length (cm) | 6.120 | 6.750 | 6.030 | 4.680 | 4.460 |
| MCF | 1.127 | 1.233 | 1.120 | 1.050 | 1.182 |
| HSI | 0.060 | 0.059 | 0.064 | 0.053 | 0.049 |
| Shrimp-Antenna Length Ratio | 1.000 | 0.996 | 0.718 | 0.478 | 0.467 |
|  |  |  |  |  |  |
| **OI.STA** | **D43** | **D50** | **D57** | **D64** | **D71** |
| Weight (g) | 3.110 | 4.728 | 5.532 | 8.240 | 10.004 |
| Length (cm) | 6.100 | 7.580 | 7.940 | 9.040 | 9.580 |
| Hepatopancreas weight (g) | 0.186 | 0.274 | 0.290 | 0.418 | 0.532 |
| Antenna Length (cm) | 5.990 | 4.110 | 3.310 | 5.430 | 4.170 |
| MCF | 1.370 | 1.086 | 1.105 | 1.115 | 1.138 |
| HSI | 0.060 | 0.058 | 0.052 | 0.051 | 0.053 |
| Shrimp-Antenna Length Ratio | 0.982 | 0.542 | 0.417 | 0.601 | 0.435 |
|  |  |  |  |  |  |
| **OI.STB** | **D43** | **D50** | **D57** | **D64** | **D71** |
| Weight (g) | 2.034 | 2.808 | 4.686 | 7.424 | 11.250 |
| Length (cm) | 5.520 | 6.260 | 7.540 | 8.820 | 9.860 |
| Hepatopancreas weight (g) | 0.179 | 0.175 | 0.303 | 0.402 | 0.539 |
| Antenna Length (cm) | 6.390 | 5.380 | 4.220 | 4.410 | 4.940 |
| MCF | 1.209 | 1.145 | 1.093 | 1.082 | 1.174 |
| HSI | 0.088 | 0.062 | 0.065 | 0.054 | 0.048 |
| Shrimp-Antenna Length Ratio | 1.158 | 0.859 | 0.560 | 0.500 | 0.501 |
|  |  |  |  |  |  |
| **OI.STC** | **D43** | **D50** | **D57** | **D64** | **D71** |
| Weight (g) | 2.498 | 3.352 | 7.482 | 10.022 | 13.706 |
| Length (cm) | 5.860 | 6.560 | 8.900 | 9.680 | 10.540 |
| Hepatopancreas weight (g) | 0.156 | 0.200 | 0.426 | 0.504 | 0.682 |
| Antenna Length (cm) | 6.150 | 6.760 | 6.410 | 3.980 | 6.290 |
| MCF | 1.241 | 1.187 | 1.061 | 1.105 | 1.171 |
| HSI | 0.062 | 0.060 | 0.057 | 0.050 | 0.050 |
| Shrimp-Antenna Length Ratio | 1.049 | 1.030 | 0.720 | 0.411 | 0.597 |

**Table S10.** Stocking, stocking density, termination count, termination density, mortality rate and daily mortality rate data.

|  | **SIS.ST1** | **SIS.ST2** | **SIS.ST3** | **SIS.STA** | **SIS.STB** | **SIS.STC** | **OI.ST1** | **OI.ST2** | **OI.ST3** | **OI.STA** | **OI.STB** | **OI.STC** |
| --- | --- | --- | --- | --- | --- | --- | --- | --- | --- | --- | --- | --- |
| STOCK COUNT | 1999 | 2002 | 2006 | 2006 | 2000 | 2003 | 1943 | 1943 | 1944 | 1943 | 1945 | 1945 |
| STOCK DENSITY | 0.16 | 0.181 | 0.188 | 0.161 | 0.181 | 0.188 | 0.202 | 0.202 | 0.202 | 0.202 | 0.202 | 0.202 |
| TERMINATION COUNT | 1265 | 1033 | 1436 | 1229 | 1193 | 1251 | 1287 | 1229 | 1175 | 1191 | 1164 | 1055 |
| TERM. DENSITY | 1.437 | 1.675 | 1.481 | 1.535 | 1.667 | 1.431 | 1.541 | 1.518 | 1.782 | 1.57 | 1.566 | 1.764 |
| MORTALITY | 16.7% | 36.6% | 22.5% | 26.5% | 28.0% | 24.1% | 19.1% | 22.9% | 25.3% | 24.8% | 26.2% | 30.1% |
| DAILY MORTALITY | 0.39% | 0.85% | 0.52% | 0.62% | 0.65% | 0.56% | 0.45% | 0.53% | 0.59% | 0.58% | 0.61% | 0.70% |
